# Increased Medical Comorbidities in Essential Tremor—A Wake-up Call?

**DOI:** 10.36469/001c.38905

**Published:** 2022-11-01

**Authors:** Elan D. Louis

**Affiliations:** 1 Department of Neurology University of Texas Southwestern Medical Center, Dallas

**Keywords:** essential tremor, comorbidity, health economics, costs, healthcare utilization

I read with great interest the article by Dai et al^1^ and was intrigued by data that demonstrated significant increases in the prevalence of numerous medical comorbidities in patients with essential tremor (ET). The authors reported that patients with ET had a higher number of comorbidities than non-ET patients (5.3 [3.2] vs 4.0 [3.3]).^1^ The study had numerous strengths. First, the team used the *International Classification of Diseases, Tenth Revision, Clinical Modification* (ICD-10-CM) code G25.0, which is more specific for ET and better able to cleanly remove patients with other tremor disorders than the prior ICD-9 code. Thus, the risk of diagnostic misclassification is substantially lower than in the past. Second, a non-ET comparison cohort was carefully created using 1:1 matching on numerous potential confounders, including age, sex, payer type, first 3 digits of zip code, and index month. This provides reassurance that the groups are comparable. Third, the sample size in both the ET and the non-ET comparison groups was more than 5000.

Prevalence values were higher in the ET group than in the comparison group for 27 of the 30 comorbidities for which data were presented. Several of these are well-established associations, such as dementia, depression, and anxiety. However, there were numerous other associations. The sample size was large, and it is conceivable that differences could have emerged that, while statistically significant, were not clinically meaningful. Yet the difference in prevalence between the ET and the non-ET group was more than 10% for several comorbidities: hypertension, pain disorders, hyperlipidemia, fatigue and sleep-related disorders, and chronic kidney disease.

This increased association of ET with numerous other comorbidities is noteworthy because it goes against the canonical thinking that ET is not associated with an increased risk of nonpsychiatric comorbidities, which does not appear to be based on any rigorous published data. To my knowledge, no studies, other than that of Handforth and Parker,^2^ have carefully compared the prevalence of comorbidities in ET patients with matched controls. While clinical neurologists have not noted any glaring patterns of increased comorbidity in patients with ET, quite frankly, they have not been looking. Furthermore, it would be relatively easy in clinical practice to miss a 10% or 20% difference in prevalence across 100 patients with ET vs those with another condition who were seen over a multi-year period. Additionally, what would be the appropriate comparison group? Most neurologists care for patients with neurological conditions, and most movement-disorder neurologists care for many patients with Parkinson’s disease. It is unlikely that a clinic could yield an appropriate comparison group. Clearly, the clinic is not the proper venue in which to adequately assess such associations.

In response to the data presented by Dai et al,^1^ there is a tendency to search for other data to provide corroborating evidence. In a study of more than 5 million US veterans, patients with ET were more likely than those without ET to have numerous comorbidities.^2^ Patients with ET were, in fact, more likely to have many of the comorbidities reported by Dai et al,^1^ including hypertension, obesity, hyperlipidemia, diabetes mellitus, ischemic heart disease, and inflammatory bowel disease.^2^ In the veteran study, post-traumatic stress disorder, anxiety, and depression were the most common psychiatric diagnoses in ET patients, with the odds ratio exceeding 2.^2^ The authors of the veterans’ study argued that post-traumatic stress disorder, anxiety, and depression, which they found were strongly associated with ET, are known risk factors for poor health habits and tobacco and alcohol use, which in turn are risk factors for vascular disease, with further negative health consequences for multiple organ systems.^2^

Neurologists are slowly waking up to the understanding that ET, long characterized and referred to as a “benign” condition,^3^ is both a chronic and progressive disease, likely neurodegenerative in nature,^4^ which is associated with increased risks of other neurodegenerative diseases, such as Parkinson’s disease^5^ and dementia.^6^ In turn, these conditions are associated with a significant burden of psychiatric comorbidity,^4^ and now, perhaps through this connection, may be associated with a significantly higher burden of medical comorbidity.

While the effect of this increased comorbidity in ET still needs to be understood clinically, Dai et al^1^ point out, from a health economics vantage point, that there is an associated increase in healthcare resource utilization and costs among adults with ET. Given the high prevalence of ET,^7^ affecting 2.2% of the entire US population,^8^ the extent of individual-level medical spending would be greatly magnified to a population level. A recent health economics study estimated that, across the population, aggregated additional spending attributable to ET among Medicare beneficiaries was between $1.5 billion and $5.4 billion per year.^9^ Research such as that presented by Dai et al^1^ begins to provide insights into the potential sources of such spending.

Elan D. Louis, MD, MS
